# Persistent hyperglycemia is a useful glycemic pattern to predict stroke mortality: a systematic review and meta-analysis

**DOI:** 10.1186/s12883-021-02512-1

**Published:** 2021-12-14

**Authors:** Duanlu Hou, Ping Zhong, Xiaofei Ye, Danhong Wu

**Affiliations:** 1 Department of Neurology, Shanghai Fifth People’s Hospital, Fudan University, 801 Heqing Road, Minhang District, Shanghai, 200240 China; 2 Department of Neurology, Shidong Hospital of Yangpu District, Shanghai, China; 3 Department of Health Statistics, Naval Military Medical University, Shanghai, China

**Keywords:** Persistent hyperglycemia, Stroke, Mortality, meta-analysis

## Abstract

**Background:**

Glycemic patterns have been reported to be prognostic factors for stroke; however, this remains to be further evaluated. This meta-analysis aimed to evaluate the usefulness of glycemic patterns such as persistent hyperglycemia (PH) including short duration and long duration PH (SPH; LPH), admission hyperglycemia (AH), short-duration hyperglycemia (SH), and persistent normoglycemia (PN) in predicting stroke prognosis using published results.

**Methods:**

Major scientific databases including but are not limited to PubMed, EMBASE, Web of Science, Ovid, CNKI (Chinese National Knowledge Infrastructure), and Clinicaltrials.gov were searched till 1st March 2021 for clinical trials on the correlation between glycemic patterns and stroke outcomes. The primary outcome was defined as short-term (1- or 3-month) post-stroke mortality, and the secondary outcome was post-stroke hemorrhage at 6 months.

**Results:**

Ten studies involving 3584 individuals were included in the final analysis. In subgroup analyses, PH patients with no history of diabetes had increased post-stroke mortality (odds ratio [OR]: 4.80, 95% CI: 3.06–7.54) than patients with no PH; and patients with glucose levels > 140 mg/dl had greater mortality (OR: 5.12, 95% CI: 3.21–8.18) than those with glucose levels < 140 mg/dl; compared with AH patients, PH patients had increased short-term mortality (OR: 0.31, 95% CI: 0.16–0.60). In the prediction of stroke mortality among patients without diabetes, SPH (OR: 0.28, 95%CI: 0.12–0.69) seemed to be more related to increased mortality than LPH (OR: 0.35, 95% CI: 0.14–-0.90).

**Conclusions:**

PH, especially SPH, could predict increased post-stroke mortality in non-diabetic patients. The rank of individual glycemic patterns in predicting stroke mortality in non-diabetic patients was SPH > LPH > AH > PN.

**Supplementary Information:**

The online version contains supplementary material available at 10.1186/s12883-021-02512-1.

## Background

Stroke, especially ischemic stroke, has a high incidence of mortality and morbidity [1]. Many factors, such as age, National Institutes of Health Stroke Scale (NIHSS) score at admission, infarct size, history of diabetes mellitus (DM), hypertension, and blood glucose level, have been used to predict short-term post-stroke mortality [2, 3]. A higher blood glucose level at admission predicts worse short-term stroke outcomes, such as increased mortality and hemorrhagic transformation (HT) in patients with ischemic stroke [4–7]. Persistent hyperglycemia (PH), defined as a hyperglycemic state with blood glucose levels > 140 or 150 mg/dl measured at admission and at a random time point within a duration (≥ 24 h, i.e., 48–72 h [8]) since admission [9, 10], can predict poor functional outcomes and increased HTs in patients with stroke with or without DM [9, 10]. In addition, PH can be divided into two subgroups: short duration PH (SPH) and long-duration PH (LPH); SPH is defined as a high glucose status at the time of admission and at a random time after admission, with the random time after admission being within 24 h after admission, and LPH is defined as a high glucose status at the time of admission and at a random time after admission, with the random time after admission being greater than 24 h after admission. However, other studies found no significant difference in this prediction between persistent hyperglycemic and normoglycemic states in patients with stroke [8, 11]. Hyperglycemia of long duration (more than 1 d after admission) can better predict worse stroke outcomes than a single-point hyperglycemia after stroke onset (SH, defined as hyperglycemia only at 24 h after stroke onset) [9, 10]) or at admission (AH, which was defined as hyperglycemia at admission) [8, 10, 12–14]. Apart from the studies mentioned above, few studies have investigated the efficacy of PH, AH, and SH in predicting post-stroke outcomes, and the superiority of PH over SH and AH. This meta-analysis aimed to answer this question by summarizing currently available results on the efficacy of PH, AH, and SH in predicting stroke outcomes, which will contribute to the selection of treatments to improve post-stroke prognosis.

## Methods

### Literature search

This study was reported following the Preferred Reporting Items for a Systematic Review and Meta-analysis (PRISMA) [15] and Meta-analysis of Observational Studies in Epidemiology (MOOSE) [16] guidelines (see Supplemental Materials). PubMed, Embase, Web of Science, Ovid, China National Knowledge Infrastructure, and ClinicalTrials. gov were searched for relevant studies published or registered before March 1, 2021. Studies on the correlation between hyperglycemia, especially persistent hyperglycemia, and stroke outcomes were included for further analysis. Key words used for searching included persistent hyperglycemia (such as “persistent hyperglycemia” or “hyperglycemia”), admission hyperglycemia (such as “hyperglycemia at admission” or “admission hyperglycemia”) were combined with key terms related to stroke outcomes (such as “stroke outcomes” or “stroke prognosis”). No language restrictions were imposed. The exact search strategy and rationale are shown in Supplementary file 1. Additional articles were obtained from the reference lists of the articles identified in the initial search.

### Selection criteria

The inclusion criteria were as follows: 1) cohort, case-control, and cross-sectional studies including unpublished studies that focused on correlations between AH, SH, PH, PN, and stroke outcomes, and must include PH; 2) stroke (ischemic and hemorrhagic stroke) must be well confirmed on either magnetic resonance imaging or computed tomography. Exclusion criteria included: 1) studies without a clear definition of outcomes (i.e., mortality and HT) or glycemic patterns; 2) studies aimed at insulin therapy or other therapies, or glucose monitoring for stroke patients with abnormal glucose levels; 3) studies, including unpublished studies, lacking sufficient data for analysis. Cohort, case-control, and cross-sectional studies that examined the correlation between blood glucose levels and stroke were included for further analysis. Two reviewers (D Hou and D Wu) independently reviewed the title, abstract, and full text of each article, and details of their results were entered into a data extraction form. A third reviewer (P Zhong) checked and approved the study. When data were missing, the corresponding authors of those studies were contacted through e-mail for further information (mainly by D Hou). If the corresponding author could not provide the missing data, the study was excluded. The primary outcome was 30-day or 3-month mortality (or mRS = 6) of patients with stroke belonging to the four glycemic patterns, namely AH, SH, PH, and persistent normoglycemia (PN). The secondary outcome was HT or re-bleeding at 6 months.

### Data extraction

Two investigators independently extracted the data and entered them into the data extraction form. The following information was recorded: the first author, publishing date, study design/study name, geographical location, population/ethnicity, time of baseline survey, sample population, definition, sample size, sex, summary statistics (using a standardized extraction form), and degree of adjustment for potential confounders (Table 1).

**Table 1:** Characteristics of included studies

First author, year of publication	Name of study or source of participants	Years of sample collection	Definition of hyperglycemia (glucose)	Definition of persistent hyperglycemia (glucose measured at)	Patterns of hyperglycemia	Outcome (mortality)	Participants	Male	Numbers of 30-d death				Numbers of short-term cerebral hemorrhage			
Mi D et al. 2018 [14]	Tiantan Hospital	2014–2016	≧ 7.8 mmol/L	0^b^, 24^b^ hours	[1–4]^a^	30-day	91 Chinese	65	1	1	12	1	1	2	13	2
Li G et al. 2018	Qilu Hospital	2008–2009	Admission: ≧ 7.8 mmol/L; In-patient: ≧ 6.1 mmol/L	0 h; 1, 2, 3, 5, 7 days	[1–4]	3-month	150 Chinese	73	1	5	2	4	NA			
Yong M et al. 2008 [10]	ECASS-II	1996–1998	≧ 140 mg/dl	0, 24 h	[1–4]	30-day	587 Westerners (nondiabetic)	100	8	14	14	20	44	28	18	163
Yong M (2) et al. 2008 [10]	ECASS-II						161 Westerners (diabetic)		4	2	9	2	9	6	42	10
Feng W et al. 2012 [17]	NA	2012	≧ 150 mg/dl	Duration: 72 h	low/high	3-month	135 Westerners	31	Low:18; high:23				NA			
Wu T et al. 2017 [8]	HICHS	2005–2016	≧ 8.0 mmol/L	24, 24–72 h	[1–4]	6-month	576 Westerners	342	36	5	70	26	NA			
Merlino G et al. 2020 [9]	Udine University Hospital	2015–2019	> 140 mg/dL	0, 24 h	[1–4]	3-month	200 Westerners	101	12	3	4	21	9	2	4	110
Ntaios G et al. 2010	ASTRAL	2003–2009	≧ 8.0 mmol/L	0, 24–48 h	Low/high	3-month	421 Westerners	240	Low:124; high:60				NA			
Fuentes B et al. 2010 [12]	GLIAS	2002–2003	≧ 155 mg/dl	2 times in 0–48 h	[1–3]	3-month	476 Westerners	234	[1–3]				8, 15, 28			
Hou D et al. 2021 [18]	Shanghai Fifth People’s Hospital	2017–2020	≧ 11.1 mmol/L	4 times a day for 7–14 days	With/without PH	1-month	200 Chinese	105	With: 11; without:6				NA			

*Abbreviations*: *ECASS-II* European Cooperative Acute Stroke Study-II, *NA* Not available, *HICHS* Helsinki Intracranial Cerebral Hemorrhage Study, *ASTRAL* Acute STroke Registry and Analysis of Lausanne, *GLISAS* Glycemia in Acute Stroke, *PH* Persistent hyperglycemia

^a^ [1–4]: four patterns of hyperglycemia; 1 for admission hyperglycemia; 2 for short-duration hyperglycemia; 3 for persistent hyperglycemia; and 4 for persistent normoglycemia; ^b^ 0 h: at admission; and 24 h: 24 h after the patient admission

### Quality assessment

Study quality was evaluated using the Newcastle-Ottawa Scale (NOS) for cohort and case-control studies (see Supplemental Materials). The quality of studies was determined by examining their compliance to the selection criteria, comparability of cases and controls, exposure, and outcome assessments. For cross-sectional studies, quality was assessed using the NOS modified for this type of study [19]. Overall, a score of ≥5 indicated adequate quality for inclusion in this meta-analysis.

### Statistical analysis

The primary and secondary outcomes of the included studies were analyzed as categorical variables with the effectiveness of different treatments evaluated and interpreted with a summary odds ratio (OR) and their corresponding 95% confidence intervals (CI). Classic χ^2^ test, Q^2^, and I^2^ statistics were used to assess the magnitude of heterogeneity between the studies. The significance level was set at *P* < 0.05. In the analysis, a random-effects model was used. We assumed a priori that the meta-analysis could be affected by the variance between studies due to the different inclusion criteria, which is more appropriately addressed by a random-effect model rather than a fixed-effect model. Inverted funnel plots were used to assess the potential presence of a publication bias. All data analyses were conducted and verified using Review Manager 5.4 (Cochrane Collaboration, Oxford, UK) and Stata/SE 15.0 (Stata Corp., USA).

### Patients enrollment of the cohort study in Shanghai (or Hou et al., 2021)

Consecutive patients with either ischemic or hemorrhagic stroke were screened and selected from the Stroke Unit of Shanghai Fifth People’s Hospital between April 1, 2017, and February 1, 2020. The inclusion and exclusion criteria were the same as those reported in a previous study (see Supplemental materials) [18].

### Data availability

Details of anonymized data will be available to any qualified investigators.

## Results

**Fig. 1:**
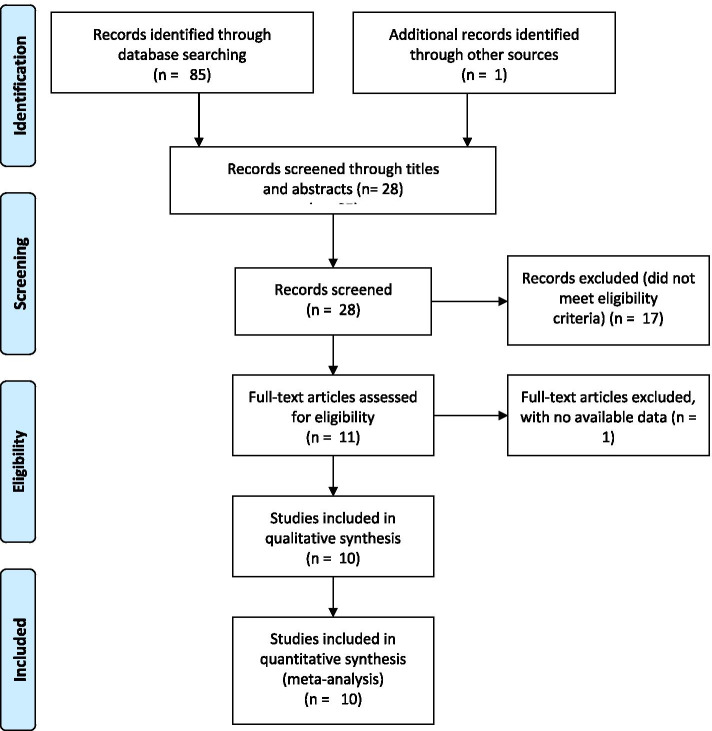
Flowchart of the study collection

The initial search found 85 articles in the databases with one additional single-centered study showing negative results on PH for stroke outcomes (the study was included after quality evaluation, NOS 7 stars) (Table S1), which is shown in the Supplementary file (Table S1, Hou, et al., 2021). The main findings and comparisons of the Shanghai study are shown in the Supplementary materials, named as a suffix: Hou et al., 2021). After carefully reading the abstracts and titles by Hou and Wu, 28 records were retained. Seventeen studies were excluded because their titles or abstracts did not meet the inclusion criteria. One was excluded because no detailed data were available. Full texts of 10 records [8–12, 14, 17, 20, 21] (one study [10] was regarded as two records because it included two groups of the population, one was on DM patients and the other on non-DM patients) were carefully evaluated by Dr. Hou, Wu, and Zhong. A total of 3584 patients were included in the final meta-analysis, as shown in Fig. 1 and Table 1.

### Overall comparisons of the primary and secondary outcomes between PH and non-PH, AH and non-AH, SH and non-SH, and PN and non-PN patients: a need for subgroup analysis

**Fig. 2:**
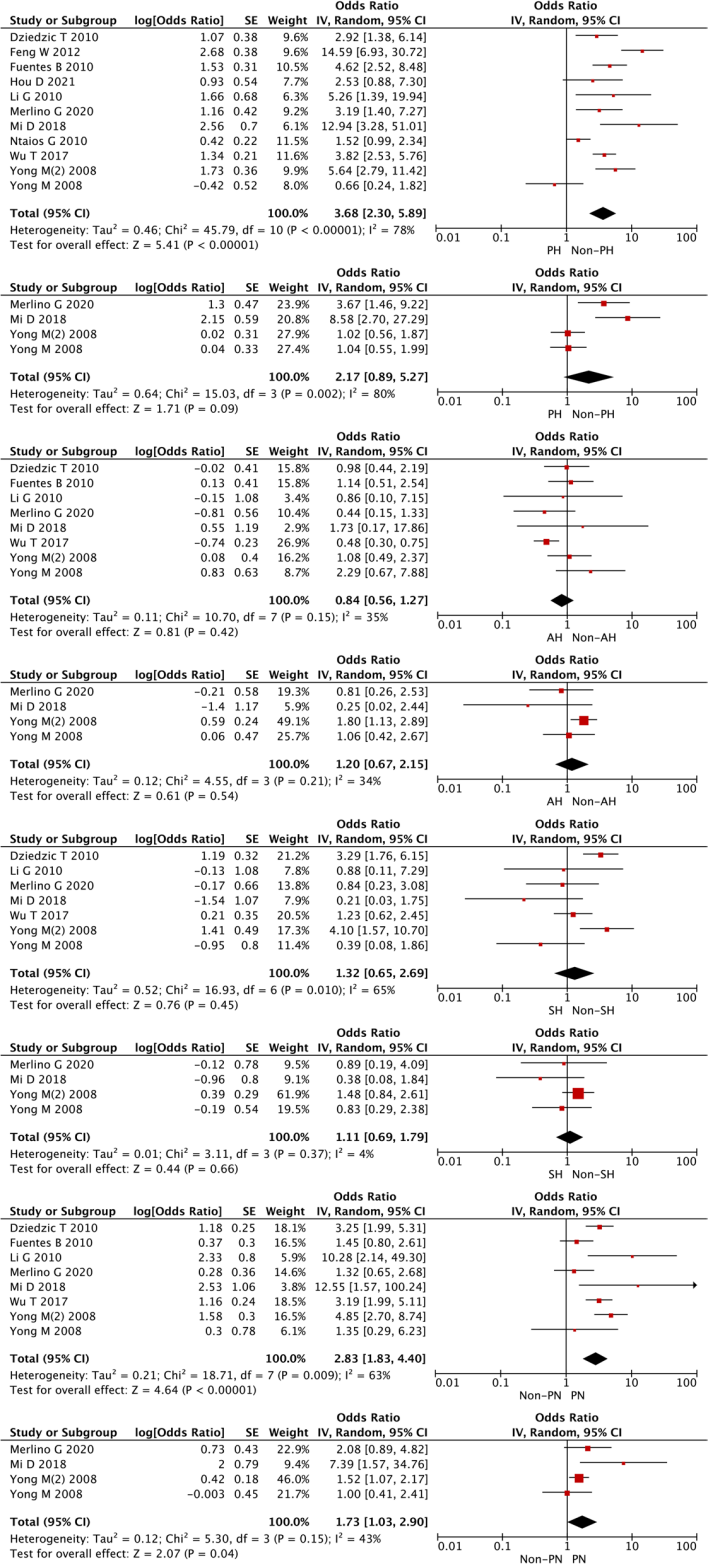
Forest plots of PH, AH, SH, non-PN for poststroke mortality (**A**, **C**, **E**, **G**), and poststroke hemorrhage prediction (**B**, **D**, **F**, **H**)

The correlation between AH, SH, PH, or PN and stroke outcomes is shown in Fig. 2. In 10 cohort studies, 3275 patients were included to assess the correlation between the primary outcome and PH in both PH and non-PH patients (Fig. 2A). Compared with PH patients, non-PH patients tended to have lower mortality (OR: 3.68, 95% CI 2.30–5.89), which seemed to have a protective role. However, there was significant heterogeneity among these studies (I^2^ = 78%, *P* < 0.00001). We then removed the data from Yong from the original analysis and performed the statistical analysis again (Fig. S4), and found that the heterogeneity of the data did not decrease significantly (I^2^ = 0.69, *P* = 0.003), which suggests that the determining factor leading to heterogeneity is not the presence or absence of the study by Yong (2008), but probably the different internal design of each study. In three cohort studies, 1079 patients were included to assess the correlation between PH and the secondary outcome in both PH and non-PH patients (Fig. 2B) with significant heterogeneity (I^2^ = 80%, *P* = 0.002). When the correlation between short-term mortality, hemorrhagic rate, and AH or SH was analyzed, no significant difference in short-term mortality or hemorrhagic rate was found between AH and non-AH patients, or between SH and non-SH patients (Fig. 2C-D). In contrast, a negative correlation was found between PN and mortality as well as hemorrhage (OR: 2.83, 95% CI: 1.83–4.40 for mortality, I^2^ = 0.63, *P* = 0.009; OR: 1.73, 95% CI: 1.03–2.90 for hemorrhage, I^2^ = 0.43, *P* = 0.15).

### Subgroup analysis of the correlations in DM or non-DM patients: primary and secondary outcomes

Based on the characteristics of the included studies, we grouped them based on a number of variables (i.e., presence of DM, duration of PH, and different definitions of hyperglycemia). It was found that PH was positively correlated with adverse outcomes (i.e., death) in stroke patients without a history of DM (OR: 4.80, 95% CI: 3.06–7.54) compared with patients without PH. The heterogeneity of these studies was relatively small (I^2^ = 24%, *P* = 0.27). These results suggest that the presence or absence of DM is a particularly critical confounding factor that affects the efficacy of PH in predicting post-stroke mortality. In four studies, 249 patients were included to assess the correlation between AH or PH and primary outcomes (Fig. 3.1.1). It was found that AH patients had lower post-stroke mortality (OR: 0.31, 95% CI: 0.16–0.60) than PH patients in those without DM, whereas no significant difference was found in patients with DM (Fig. 3.1.2), suggesting that PH had a significant impact on stroke outcome in non-diabetic patients. Short duration (< 24 h) of PH or SPH was correlated with higher stroke mortality (OR: 0.28, 95% CI: 0.12–0.69) than long duration (> 24 h) of PH or LPH (OR: 0.35, 95%CI: 0.14–0.90) in stroke patients without DM. Both SPH and LPH were better than AH in predicting stroke mortality. Therefore, the rank of the efficacy of glycemic patterns in predicting stroke mortality in nondiabetic patients was SPH > LPH > AH > PN.

**Fig. 3:**
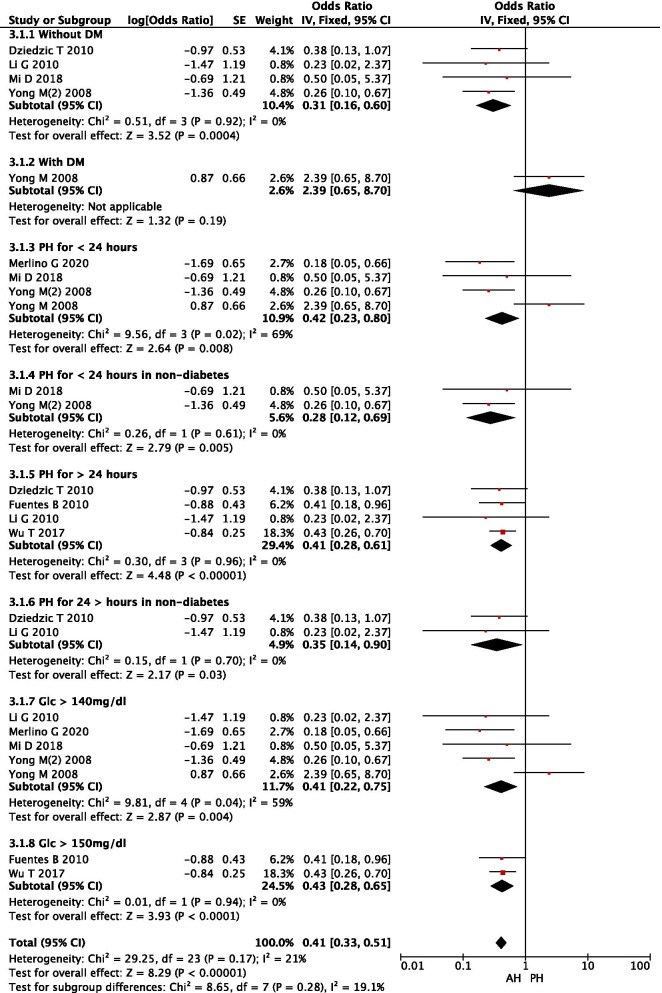
Forest plot of subgroup analysis on AH or PH for predicting post-stroke mortality

**Fig. 4:**
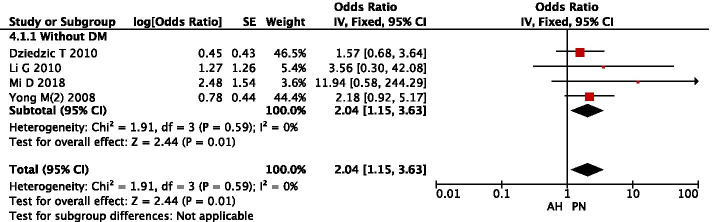
Forest plot of subgroup analysis on AH or PN for predicting post-stroke mortality

In four studies, 857 patients were included to assess the correlation between AH or PN and primary outcomes (Fig. 4). No heterogeneity was found between these studies (I^2^ = 0%, *P* = 0.59), and PN patients had the lower post-stroke mortality (OR: 2.04, 95% CI: 1.15–3.63) than AH patients in those without DM.

### Subgroup analysis of the correlations in patients with PH < 24 h vs > 24 h

**Fig. 5:**
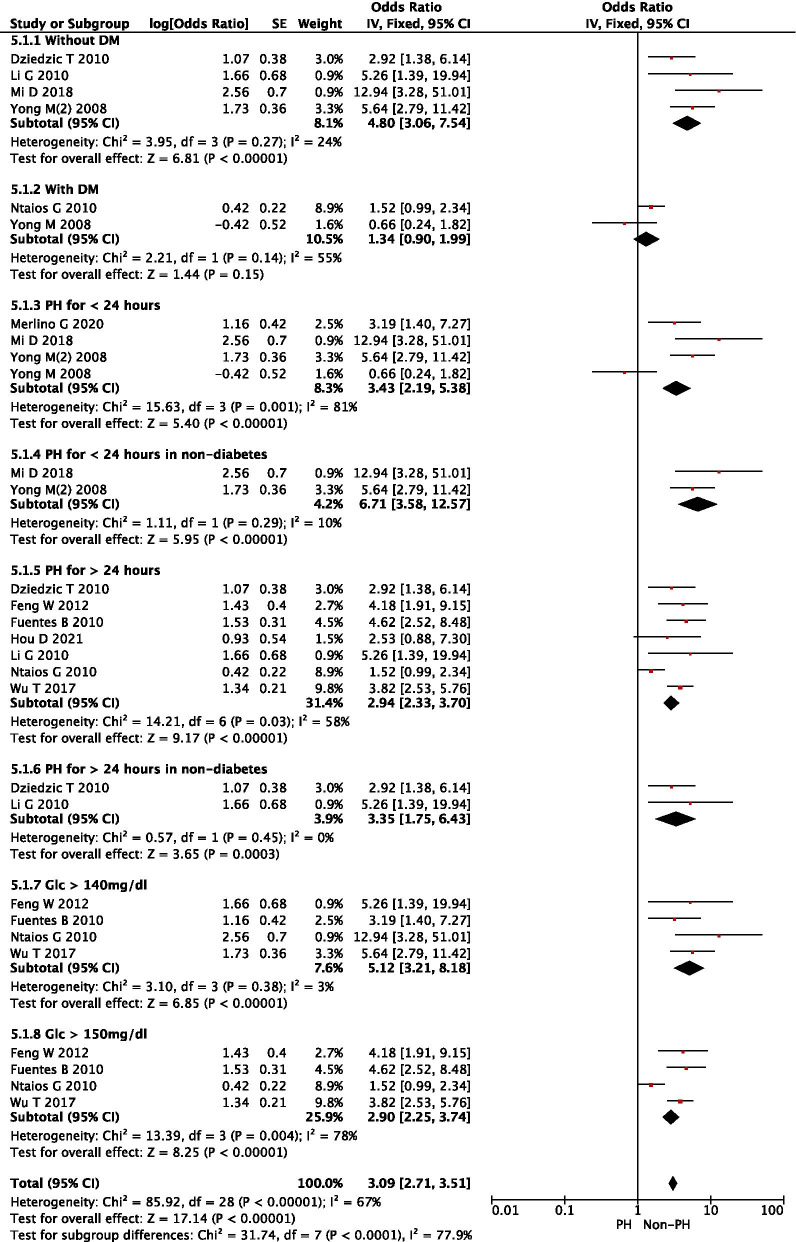
Forest plot of subgroup analysis on PH for predicting post-stroke mortality

In addition, PH < 24 h led to increased stroke mortality (OR: 6.71, 95%CI: 3.58–12.57) compared with PH > 24 h (OR: 3.35, 95% CI: 1.75–6.43) in non-diabetic stroke patients. It seemed that a short duration of 24 h of PH was better at predicting post-stroke mortality than a long duration of PH (Fig. 5).

### Subgroup analysis of the correlations in patients with glucose levels > 140 or 150 mg/dl

Furthermore, the total OR for post-stroke mortality was 5.12 (95% CI: 3.21–8.18) in non-PH patients with glucose (GLC) levels > 140 mg/dl (7.8 mmol/L), and no heterogeneity was found (I^2^ = 3%, *P* = 0.38). However, the OR was 2.90 (95% CI: 2.25–3.74) in non-PH patients with GLC levels > 150 mg/dl, and significant heterogeneity was present. It is clear from the analysis here that caution is needed in defining hyperglycemia because a higher threshold of glucose level may lose patients who should be actively treated to gain favorable outcomes. (Fig. 5).

### Publication bias and Egger’s test

**Fig. 6:**
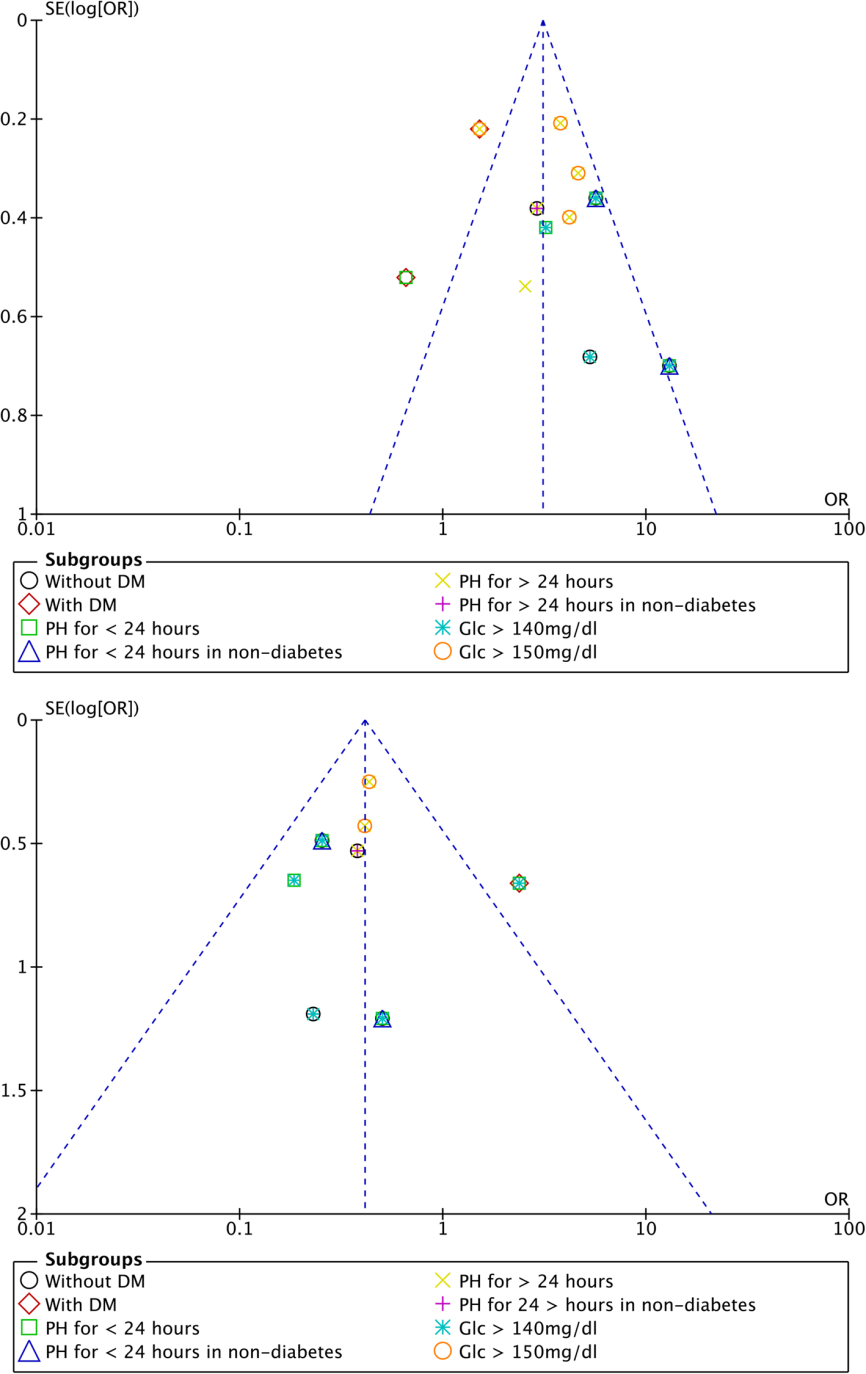
Funnel plots of the subgroup analysis of PH and non-PH (upper), and AH and PH (lower) for post-stroke mortality

Results of the Egger’s test for mortality between PH and non-PH groups (*P* = 0.584) in the general population, between the PH and non-PH groups without DM (*P* = 0.419), between the AH and PH groups without DM (*P* = 0.888), and mortality between the AH and PN groups without DM (*P* = 0.418) suggested that no publication bias was found among the included studies (Fig. 6 for two main subgroup analyses, Fig. S1, and Fig. S2, Egger).

## Discussion

This study analyzed the correlation between hyperglycemic patterns and stroke mortality. It was found that PH could predict worse stroke outcomes in non-diabetic patients. The efficacy of glycemic patterns in predicting poor stroke outcomes in nondiabetic patients was ranked as SPH > LPH > AH > PN. No conclusion on SH was reached owing to the lack of sufficient studies for analysis. The presence of DM is an important confounder. In populations with DM, heterogeneity was observed, and no difference was found in the efficacy of glycemic patterns in predicting stroke mortality and their ranking (details are shown in the Results section).

Over 50% of patients within each stroke subtype had glucose levels greater than 6.0 mmol/l on admission [22]. Post-stroke hyperglycemia not only worsens functional outcomes and vascular perfusion [23], but also leads to spontaneous intracerebral hemorrhage, impaired executive functions [24], and death [25] in stroke patients, especially in patients with large vessel occlusion [26, 27]. Blood glucose levels seem to decline within the first 24 h after stroke onset [28], but they rise again after approximately 24–88 h, regardless of the presence of DM [29, 30]. PH with high blood glucose levels lasting for 24 h or more seems to have a controversial effect on stroke outcomes [11, 13], which requires further analysis to confirm the efficacy of different glycemic patterns in predicting post-stroke mortality. The overall analysis of the general population with stroke showed a significant difference that PH patients had increased post-stroke mortality than non-PH patients and a trend that PN patients had decreased post-stroke mortality and hemorrhagic rate than non-PN patients.

A systematic review [31] found that acute hyperglycemia predicts an increased risk of in-hospital mortality after ischemic stroke in non-diabetic patients and an increased risk of poor functional recovery in non-diabetic stroke survivors, which implies the impact of high glucose levels on stroke mortality. Thus, hyperglycemic status in non-diabetic patients is more closely related to the prognosis of ischemic stroke. There are a couple of reasons for the low prognostic efficacy of AH and PH in patients with DM. First, there are many confounding factors, including the presence of multiple complications (such as kidney and heart diseases) [32] and glucose-lowering therapies in elderly people with long-term DM (mainly type 2 DM), that influence the prognostic efficacy. Second, the super-chronic mild hyperglycemic state (in which blood glucose levels are not that high due to the effect of glucose-lowering drugs) does not have a significant impact on the short-term prognosis; instead, this life-long mild hyperglycemic state might affect outcomes decades later.

A correlation between stress hyperglycemia and vascular damage has also been reported [31]. Hyperglycemia was unable to predict stroke mortality independently. However, when NIHSS was removed from the multivariate model, stress hyperglycemia became an independent predictor of in-hospital mortality along with age, atrial fibrillation (AF), diastolic blood pressure (DBP), and log-triglyceride (TG) levels [33]. This suggests that hyperglycemia in non-diabetic patients reflects the severity of stroke. It is hypothesized that AH or stress hyperglycemia due to the release of stress hormones by the nervous system [34] may be a marker of stroke severity. The possible underlying mechanism is that a high level of glucose in the brain leads to cell death through the activation of hexokinase II [35, 36]. The negative correlation observed in the present study might be due to the difference in the definition of hyperglycemia (i.e., 126 mg/dl, not 140 mg/dl). Among the included studies, some included populations with the same disease severity [9, 10], and some studies included patients with slightly higher NIHSS scores and PH [8, 11, 12, 14, 20, 21], but the outcome was mostly worse in patients with PH. Most of the studies removed the effect of disease severity in the final multivariable analysis [8, 11, 12, 20, 21]; therefore, the effect of disease severity on disease prognosis was not decisive or significant.

SH and SPH differ in the context of this study. In general, SH is defined as the presence of hyperglycemia at a random time point within 24 h of stroke, whereas SPH is a subgroup based on PH. A persistent hyperglycemic state is theoretically a state of high blood glucose that persists over a certain period and is present at every time point during a given period. In the real world, however, a persistent hyperglycemic state is often defined by the results of two tests: a hyperglycemic state on admission to the hospital and a hyperglycemic state at a certain moment (this one moment is randomly chosen) within approximately 24 h or 24–48 h or 72 h, or longer after admission [8, 9]. The definition of PH varies between studies because of the different “real-world contexts” in which they were conducted, such as geography, but essentially, it is the detection of the hyperglycemic state at two or more random moments.

A clear definition of PH in the non-diabetic population is still under debate, and a couple of studies proposed that the possible definition may be a persistent (more than 24 h) pathological condition with GLC >  140 mg/dl that is not due to chronic insulinopenia or chronic insulin resistance [37, 38]. The duration differs among studies; some studies defined 24 h as the persistent state [9, 14] and others defined 24–48 h or 24–72 h [8], or more than 3 days. The difference in the definition of the persistent state resulted in inconsistent conclusions in the abovementioned studies. The longer the duration of the persistent state, the more negative the results [11].

Our unpublished data (Hou et al.) focused on patients with severe stroke defined by an NIHSS score > 10, and we found that patients with a hyperglycemic status could last for 2 or even 3 weeks during the 1-month follow-up period. Surprisingly, persistent hyperglycemic status was unable to independently predict 1-month mortality (see Supplemental materials Table S1-S5, Hou et al., and Table 1). Unfortunately, this study did not include a subgroup analysis of patients with and without diabetes, which might be a confounding factor.

Therefore, as far as the present conclusion is concerned, PH lasting for 24 h is perhaps the best predictor of poor prognosis in stroke patients without DM.

Our study had several limitations. First, only a small number of studies were included in the meta-analysis, which may have led to selection bias. Second, there were relatively few subgroup analyses, which did not include factors such as country, gender, and age, which might result in heterogeneity. Third, the pooled studies differed in the inclusion and exclusion criteria, the definition of hyperglycemia, short-term outcomes, and concomitant treatments. The relative risks included in the meta-analysis were not adjusted for other prognostic factors, and most published studies were included.

## Conclusions

PH, especially SPH, can predict post-stroke mortality in non-diabetic patients. The efficacy of glycemic patterns in predicting poor stroke outcomes in non-diabetic patients was ranked as SPH > LPH > AH > PN. The findings of this study indicate that random blood glucose levels should be controlled to below 140 mg/dl within 24 h for patients with acute ischemic stroke without type 2 diabetes and with admission hyperglycemia. Preventing persistent hyperglycemia (> 24 h) may reduce short-term mortality.

## Supplementary Information

**Additional file 1: Supplemental file 1.** Search strategy. **Table S1.** Quality evaluation of included studies using the Newcastle-Ottawa Quality Assessment Scale (cohort study). **Table S1.** Hou et al., 2021. Baseline characteristics of the study population and bivariate comparisons between patients with favorable and unfavorable outcomes. **Table S2.** Hou et al., 2021. Comparisons of short-term outcomes between patients with and without persistent hyperglycemia. **Table S3.** Hou et al., 2021. Baseline characteristics of the study population and bivariate comparisons between short-duration and long-duration persistent hyperglycemia groups. **Table S4.** Hou et al., 2021. Comparisons of short-term outcomes between patients with short- and long-duration persistent hyperglycemia. **Table S5.** Hou et al., 2021. Comparisons of short-term outcomes between persistent hyperglycemia patients with HbA1c < 7% and HbA1c > = 7%. **Figure S1**. Funnel plots of PH, AH, SH, non-PN for predicting post-stroke mortality and post-stroke hemorrhage [from upper left to upper right (A–B), middle left to middle right (C–D, E–F), lower left to lower right (G–H)]. **Figure S2**. egger: Egger’s test results of PH and non-PH groups in the general population, PH and non-PH groups without DM, AH and PH groups without DM, and AH and PN groups without DM for predicting post-stroke mortality. **Figure S3.** Forest plot of PH and non-PH for predicting mortality in patients with ischemic stroke. **Figure** **S4.** Revised overall comparisons between PH and non-PH, AH and non-AH, SH and non-SH, PN and non-PN groups (revised figure 2A, removed some data that may cause heterogeneity).

## Data Availability

The datasets used and/or analyzed in the present study are available from the corresponding author upon reasonable request.

## Abbreviations

PH: Persistent hyperglycemia
AH: Admission hyperglycemia
SH: Short-duration hyperglycemia
PN: Persistent normoglycemia
OR: Odds ratio
NIHSS: National Institutes of Health Stroke Scale
DM: Diabetes mellitus
HT: Hemorrhagic transformation
CNKI: China National Knowledge Infrastructure
NOS: Newcastle-Ottawa Scale
AF: Atrial fibrillation
DBP: Diastolic blood pressure
TG: Triglyceride
