# Supplementary material for: Persistent hyperglycemia is a useful glycemic pattern to predict stroke mortality: a systematic review and meta-analysis
Source: BMC Neurol. 2021 Dec 14;21:487. doi: 10.1186/s12883-021-02512-1 (PMC8670037; doi:10.1186/s12883-021-02512-1)
Supplement: Supplementary file 1 — Additional file 1: Supplemental file 1. Search strategy. Table S1. Quality evaluation of included studies using the Newcastle-Ottawa Quality Assessment Scale (cohort study). Table S1. Hou et al., 2021. Baseline characteristics of the study population and bivariate comparisons between patients with favorable and unfavorable outcomes. Table S2. Hou et al., 2021. Comparisons of short-term outcomes between patients with and without persistent hyperglycemia. Table S3. Hou et al., 2021. Baseline characteristics of the study population and bivariate comparisons between short-duration and long-duration persistent hyperglycemia groups. Table S4. Hou et al., 2021. Comparisons of short-term outcomes between patients with short- and long-duration persistent hyperglycemia. Table S5. Hou et al., 2021. Comparisons of short-term outcomes between persistent hyperglycemia patients with HbA1c < 7% and HbA1c > = 7%. Figure S1. Funnel plots of PH, AH, SH, non-PN for predicting post-stroke mortality and post-stroke hemorrhage [from upper left to upper right (A–B), middle left to middle right (C–D, E–F), lower left to lower right (G–H)]. Figure S2. egger: Egger’s test results of PH and non-PH groups in the general population, PH and non-PH groups without DM, AH and PH groups without DM, and AH and PN groups without DM for predicting post-stroke mortality. Figure S3. Forest plot of PH and non-PH for predicting mortality in patients with ischemic stroke. Figure S4. Revised overall comparisons between PH and non-PH, AH and non-AH, SH and non-SH, PN and non-PN groups (revised figure 2A, removed some data that may cause heterogeneity). [file 12883_2021_2512_MOESM1_ESM.docx]

**Supplementary Materials**

Duanlu Hou^1^, MS, Ping Zhong^2^, MD, Xiaofei Ye^3^, PhD, Danhong Wu^1^*, MD

1. Department of Neurology, Shanghai Fifth People’s Hospital, Fudan University, Shanghai, China

2. Department of Neurology, Shidong Hospital of Yangpu District, Shanghai, China

3. Department of Health Statistics, Second Military Medical University, Shanghai, China

**Supplemental file 1, Searching strategy**

Pubmed

#1 (persistent hyperglycemia) OR (persistent hyperglycemia[MeSH Terms])

(("persist"[All Fields] OR "persistance"[All Fields] OR "persistant"[All Fields] OR "persisted"[All Fields] OR "persistence"[All Fields] OR "persistences"[All Fields] OR "persistencies"[All Fields] OR "persistency"[All Fields] OR "persistent"[All Fields] OR "persistently"[All Fields] OR "persistents"[All Fields] OR "persister"[All Fields] OR "persisters"[All Fields] OR "persisting"[All Fields] OR "persists"[All Fields]) AND ("hyperglycaemia"[All Fields] OR "hyperglycemia"[MeSH Terms] OR "hyperglycemia"[All Fields] OR "hyperglycaemias"[All Fields] OR "hyperglycemias"[All Fields] OR "hyperglycemia s"[All Fields])) OR (("persist"[All Fields] OR "persistance"[All Fields] OR "persistant"[All Fields] OR "persisted"[All Fields] OR "persistence"[All Fields] OR "persistences"[All Fields] OR "persistencies"[All Fields] OR "persistency"[All Fields] OR "persistent"[All Fields] OR "persistently"[All Fields] OR "persistents"[All Fields] OR "persister"[All Fields] OR "persisters"[All Fields] OR "persisting"[All Fields] OR "persists"[All Fields]) AND "hyperglycemia"[MeSH Terms])

#2 ((stroke outcome) OR (stroke outcome[MeSH Terms])) AND ((stroke[MeSH Terms])OR (stroke))

("stroke"[MeSH Terms] OR "stroke"[All Fields] OR "strokes"[All Fields] OR "stroke s"[All Fields]) AND ("outcome"[All Fields] OR "outcomes"[All Fields]) AND ("stroke"[MeSH Terms] OR ("stroke"[MeSH Terms] OR "stroke"[All Fields] OR "strokes"[All Fields] OR "stroke s"[All Fields]))

#3 ((stroke[MeSH Terms])OR (stroke)) AND ((persistent hyperglycemia) OR (persistent hyperglycemia[MeSH Terms]))

("stroke"[MeSH Terms] OR ("stroke"[MeSH Terms] OR "stroke"[All Fields] OR "strokes"[All Fields] OR "stroke s"[All Fields])) AND ((("persist"[All Fields] OR "persistance"[All Fields] OR "persistant"[All Fields] OR "persisted"[All Fields] OR "persistence"[All Fields] OR "persistences"[All Fields] OR "persistencies"[All Fields] OR "persistency"[All Fields] OR "persistent"[All Fields] OR "persistently"[All Fields] OR "persistents"[All Fields] OR "persister"[All Fields] OR "persisters"[All Fields] OR "persisting"[All Fields] OR "persists"[All Fields]) AND ("hyperglycaemia"[All Fields] OR "hyperglycemia"[MeSH Terms] OR "hyperglycemia"[All Fields] OR "hyperglycaemias"[All Fields] OR "hyperglycemias"[All Fields] OR "hyperglycemia s"[All Fields])) OR (("persist"[All Fields] OR "persistance"[All Fields] OR "persistant"[All Fields] OR "persisted"[All Fields] OR "persistence"[All Fields] OR "persistences"[All Fields] OR "persistencies"[All Fields] OR "persistency"[All Fields] OR "persistent"[All Fields] OR "persistently"[All Fields] OR "persistents"[All Fields] OR "persister"[All Fields] OR "persisters"[All Fields] OR "persisting"[All Fields] OR "persists"[All Fields]) AND "hyperglycemia"[MeSH Terms]))

#4 (admission hyperglycemia) AND (((stroke outcome) OR (stroke outcome[MeSH Terms])) AND ((stroke[MeSH Terms])OR (stroke)))

("admission"[All Fields] OR "admissions"[All Fields]) AND ("hyperglycaemia"[All Fields] OR "hyperglycemia"[MeSH Terms] OR "hyperglycemia"[All Fields] OR "hyperglycaemias"[All Fields] OR "hyperglycemias"[All Fields] OR "hyperglycemia s"[All Fields]) AND (("stroke"[MeSH Terms] OR "stroke"[All Fields] OR "strokes"[All Fields] OR "stroke s"[All Fields]) AND ("outcome"[All Fields] OR "outcomes"[All Fields]) AND ("stroke"[MeSH Terms] OR ("stroke"[MeSH Terms] OR "stroke"[All Fields] OR "strokes"[All Fields] OR "stroke s"[All Fields])))

Web of science

#1 TS=(persistent hyperglycemia) OR TI=(persistent hyperglycemia)

#2 (TS=(stroke) OR TI=(stroke)) AND (TS=(stroke outcome) OR TI=(stroke outcome))

#3 #2 AND #1

# 4 TS=(admission hyperglycemia) AND TS=(stroke)

Ovid MEDLINE(R) <1946 to March 1, 2021>

1 ..nlpx "query=persistent hyperglycemia","desiredResults=10000","minHitsDivisor=7","permitHyponyms=NO","lowestVocabularySearchLevel=none","phrasesBroken=NO","speedWanted=NoHypos","comment=Including Related Terms","elimEnable=NO","constraintMinTerms=2"

2 ..nlpx "query=stroke","desiredResults=10000","minHitsDivisor=7","permitHyponyms=NO","lowestVocabularySearchLevel=none","phrasesBroken=NO","speedWanted=NoHypos","comment=Including Related Terms","elimEnable=NO","constraintMinTerms=2"

3 ..nlpx "query=admission hyperglycemia","desiredResults=10000","minHitsDivisor=7","permitHyponyms=NO","lowestVocabularySearchLevel=none","phrasesBroken=NO","speedWanted=NoHypos","comment=Including Related Terms","elimEnable=NO","constraintMinTerms=2"

4 ..nlpx "query=persistent hyperglycemia AND stroke AND admission hyperglycemia","desiredResults=10000","minHitsDivisor=7","permitHyponyms=NO","lowestVocabularySearchLevel=none","phrasesBroken=NO","speedWanted=NoHypos","comment=Including Related Terms","elimEnable=NO","constraintMinTerms=2"

CNKI

In this database we searched in Chinese for the terms persistent hyperglycaemia, stroke.

An article was found *LI G, Wang C: [Predictive value of hyperglycaemia in the prognosis of ischaemic cerebrovascular disease]. Journal of Shandong university (health science) 2010, 48(4):1-4.*

**Approvals, Registrations, and Patient Consents of the Cohort Study in Shanghai (or Shanghai Study) (Hou et al.)**

Consecutive patients with either ischemic or hemorrhagic stroke were screened and selected from the Stroke Unit of Shanghai Fifth People’s Hospital between April 1, 2017, and February 1, 2020. Written informed consent was obtained from all patients or their families. This study was approved by the Ethical Review Board of Shanghai Fifth People’s Hospital before recruiting patients. The inclusion and exclusion criteria were the same as those reported in a previous study [1].

1. Hou D, Wang C, Ye X, Zhong P, Wu D: Persistent inflammation worsens short-term outcomes in massive stroke patients. BMC Neurol 2021, 21(1):62.

Figure S1


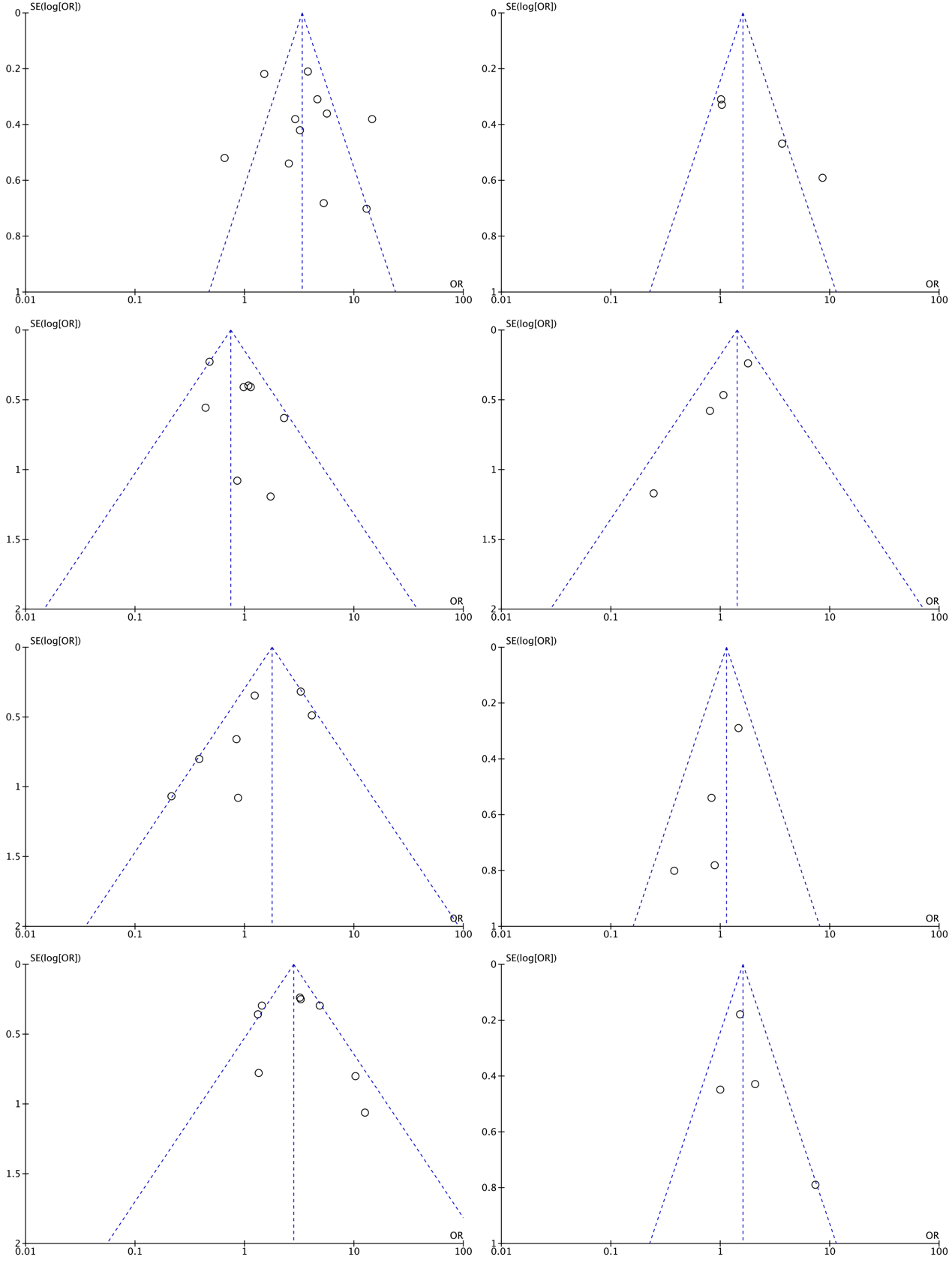


Figure S2 *see next page*


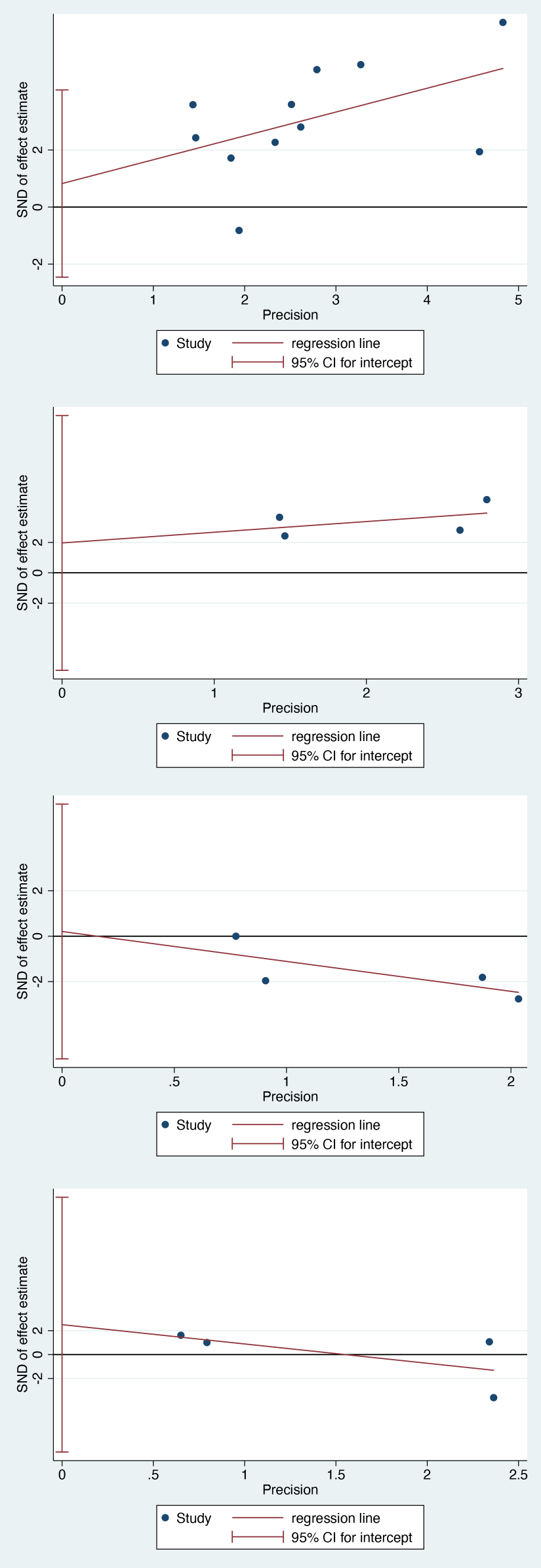


Figure S3

Figure S4

**Table S1-5 Hou et al**

| **Table S1. Baseline characteristics of the study population and bivariate comparisons between patients with favorable and unfavorable outcomes** | | | |
| --- | --- | --- | --- |
|  | Favorable functional outcome (n=102) | Unfavorable functional outcome (n=98) | *p* Value |
| Demographic data | | | |
| Male | 56 (56.1) | 49 (50) | 0.512 |
| Age ^†^ | 82 (13) | 81 (11) | 0.931 |
| Smoker | 22 (22) | 17 (17.3) | 0.526 |
| Alcohol drinker | 7 (7) | 12 (12.2) | 0.553 |
| Medical history | | | |
| Arterial hypertension | 83 (82.9) | 72 (73.4) | 0.232 |
| Diabetes mellitus | 24 (24.4) | 39 (39.8) | 0.083 |
| Atrial fibrillation | 22 (22.0) | 29 (39.8) | 0.357 |
| Coronary artery disease | 2 (2.4) | 7 (7) | 0.436 |
| Liver disease | 3 (4.9) | 8 (8.2) | 0.723 |
| Kidney disease | 2 (2.4) | 7 (7.1) | 0.436 |
| Clinical characteristics | | | |
| Systolic BP, mmHg^‡^ | 150 (132-162) | 147 (138-164) | 0.992 |
| Blood glucose, mg/dl^‡^ | 5.4 (4.6-6.3) | 6.6 (5.3-8.2) | 0.005^*^ |
| Laboratory data |  |  |  |
| HbA1c, % | 5.9 (5.5-6.6) | 6 (5.5-6.7) | 0.992 |
| Insulin, mmol/L | 59.5 (42-87) | 60.8 (42-78) | 0.834 |
| Insulin resistance | 5.8 (0-16) | 9.2 (0-20) | 0.215 |
| C peptide, ng/dl^‡^ | 0.7 (0.6-1) | 0.7 (0.6-1) | 0.611 |
| Sodium, mmol/L^‡^ | 141 (139-143) | 140 (139-142) | 0.084 |
| Potassium, mmol/L^‡^ | 4.0 (3.7-4.2) | 3.8 (3.5-4.1) | 0.144 |
| NLR at admission^‡^ | 3.3 (2.3-4.9) | 5.1 (4.0-8.3) | 0.002^*^ |
| NLR at discharge^‡^ | 3.4 (2.0-5.2) | 4.3 (2.7-6.3) | 0.028^*^ |
| Platelet, 10^9^/L^‡^ | 202 (155-239) | 193 (157-237) | 0.900 |
| LDL, mmol/L^‡^ | 2.5 (2.1-3.3) | 2.7 (1.9-3.4) | 0.837 |
| Total bilirubin, μmol/L^‡^ | 12.1 (9.6-15.9) | 15.2 (10.7-18.9) | 0.080 |
| Cystatin C, mg/L^‡^ | 1.0 (1.1-1.2) | 1.1 (1.0-1.3) | 0.922 |
| Homocysteine, μmol/L^‡^ | 14.8 (11.5-19.3) | 13.9 (11.1-19.9) | 0.990 |
| Imaging data |  |  |  |
| Cerebral ischemia | 89(87.8) | 81 (82.7) | 0.448 |
| Hemisphere | 14 (16.6) | 40 (49.3) | 0.001^*^ |
| Lobes | 22 (25) | 20 (24.6) | 0.972 |
| Brainstem/cerebellum | 29 (33.3) | 13 (16) | 0.035^*^ |
| Lacunes | 20 (22.2) | 5 (6) | 0.022^*^ |
| Cerebral hemorrhage | 10 (9.8) | 18 (18.4) | 0.205 |
| Basal ganglia | 5 (50) | 5 (28) | 0.565 |
| Lobes | 2 (25) | 12 (66) | 0.264 |
| Brainstem/cerebellum | 2 (25) | 1 (5) | 0.338 |
| Medication |  |  |  |
| rtPA | 10 (9.7) | 11 (11.2) | 0.799 |
| Intervention | 7 (7) | 5 (5.1) | 0.693 |
| In-hospital infections | | | |
| Lung infection | 32 (31.7) | 59 (60.2) | 0.002^*^ |
| Urinary tract infection | 0 (0) | 14 (14.3) | 0.011^*^ |

Abbreviations: NLR = neutrophil-to-lymphocyte ratio; BP = blood pressure; HbA1c = glycated hemoglobin 1c; LDL = low density lipoprotein; rtPA = Recombinant Human Tissue Plasminogen A.

Unless specified, values are numbers of patients (%).

**^†^**Mean (standard deviation).

**^‡^**Median (interquartile range).

^*^Statistically significant.

| **Table S3. Baseline characteristics and bivariate comparisons between short-duration and long-duration persistent hyperglycemia groups** | | | |
| --- | --- | --- | --- |
|  | Persistent hyperglycemia (1-7d) (n=102) | Persistent hyperglycemia (>= 7d) (n=98) | *p* Value |
| Demographic data | | | |
| Male | 59 (58) | 45 (45) | 0.30 |
| Age | 81 (70-85) | 83 (73-87) | 0.55 |
| Smoker | 12 (12) | 7 (29) | 0.68 |
| Alcohol drinker | 15 (15) | 0 (0) | 0.02^*^ |
| Medical history | | | |
| Arterial hypertension | 74 (73) | 72 (76) | 0.78 |
| Diabetes mellitus | 25 (24.4) | 39 (39.8) | 0.002^*^ |
| Atrial fibrillation | 22 (22.0) | 29 (39.8) | 0.99 |
| Coronary artery disease | 2 (2.4) | 7 (7) | 0.98 |
| Liver disease | 5 (4.9) | 8 (8.2) | 0.72 |
| Kidney disease | 2 (2.4) | 7 (7.1) | 0.43 |
| Clinical characteristics | | | |
| Systolic BP, mmHg^‡^ | 145 (138-160) | 152 (140-175) | 0.23 |
| Blood glucose, mg/dl^‡^ | 7.0 (6-8) | 8.8 (7.6-12.1) | 0.005^*^ |
| Laboratory data |  |  |  |
| Fasting blood glucose, mg/dl | 7.08 (5.78-8.08) | 8.57 (6.73-11.92) | 0.02^*^ |
| HbA1C, % | 6.2 (5.8-7.3) | 7.3 (6.4-8.9) | 0.01^*^ |
| Insulin, mmol/L | 63 (43-106) | 63 (42-250) | 0.75 |
| Insulin resistance | 11.8 (0-21.6) | 5.7 (0-28.8) | 0.68 |
| C peptide, | 0.73 (0.67-1.85) | 0.69 (0.47-1.91) | 0.66 |
| Sodium, mmol/L^‡^ | 140 (139-142) | 140 (138-141) | 0.36 |
| Potassium, mmol/L^‡^ | 3.8 (3.5-4.2) | 4.0 (3.6-4.2) | 0.39 |
| NLR at admission^‡^ | 4.7 (3-6 | 4.7 (3-12) | 0.59 |
| NLR at discharge^‡^ | 4.3 (3.7-5.6) | 4.7 (3.-11.3) | 0.21 |
| LDL, mmol/L^‡^ | 2.7 (1.9-3.4) | 2.8 (2.2-3.5) | 0.74 |
| Total bilirubin, μmol/L^‡^ | 12.7 (11.1-16.4) | 11.8 (8.3-16.8) | 0.29 |
| Cystatin C, ng/dl^‡^ | 1.2 (1-1.4) | 1.2 (0.9-1.8) | 0.68 |
| Homocysteine, μmol/L^‡^ | 14.4 (11.9-18.5) | 11.8 (9.5-17.8) | 0.19 |
| Imaging data |  |  |  |
| Cerebral ischemia | 97 (97) | 80 (82) | 0.01 |
| Hemisphere | 37 (39) | 40 (48) | 0.30 |
| Lobes | 6 (6) | 10 (13) | 0.33 |
| Brainstem/cerebellum | 25 (26) | 7 (9) | 0.16 |
| Lacunes | 9 (9) | 7 (9) | 0.82 |
| Basal ganglia | 19 (19) | 17 (21) | 0.87 |
| Hemorrhagic transformation | 13(13) | 18(22) | 0.32 |
| Cerebral hemorrhage | 3 (3) | 18 (18) | - |
| Basal ganglia | 3 (100) | 10 (60) | - |
| Lobes | - | 8 (40) | - |

Abbreviations: NLR = neutrophil-to-lymphocyte ratio; BP = blood pressure; LDL = low density lipoprotein; HbA1c = glycated hemoglobin 1c.Unless specified, values are numbers of patients (%).**^‡^**Median (interquartile range).^*^Statistically significant.

| **Table S4. Comparisons of short-term outcomes between patients with short- and long-duration persistent hyperglycemia** | | | | | |
| --- | --- | --- | --- | --- | --- |
| Short-term outcomes | Persistent hyperglycemia | | *p* value | *OR* | *AR* |
|  | Short-duration | Long-duration |  |  |  |
| Pulmonary infection | 27 (66) | 51 (52) | 0.27 | 1.3 | 14 |
| Urinary tract infection | 7 (16) | 24 (24) | 0.40 | 0.7 | -8 |
| 1-month mortality | 7 (16) | 21 (21) | 0.61 | 0.8 | -5 |
| 1-month mRS | 4 (4-9)**^†^** | 4 (4-9)**^†^** | 0.87 | - | - |
| Abbreviations: mRS = modified Rankin scale; RR = relative ratio; AR = attributable ratio.  Unless specified, values are *p* value in non-parametric tests and values are numbers of patients (%).  **^†^**Median (interquartile range).  ^*^Statistically significant.   \| **Table S5. Comparisons of short-term outcomes between persistent hyperglycemia patients with HbA1c<7% and HbA1c>=7%** \| \| \| \| \| \| \| --- \| --- \| --- \| --- \| --- \| --- \| \| Short-term outcomes \| Persistent hyperglycemia \| \| *p* value \| *OR* \| *AR* \| \| HbA1C<7% \| HbA1C>=7% \| \| Pulmonary infection \| 17 (57) \| 15 (58) \| 0.90 \| 0.9 \| -1 \| \| Urinary tract infection \| 5 (17) \| 5 (17) \| 1.0 \| 1 \| 0 \| \| 1-month mortality \| 3 (10) \| 5 (17) \| 0.69 \| 0.6 \| -7 \| \| 1-month mRS \| 4 (4-9)**^†^** \| 4 (4-9)**^†^** \| 0.95 \| - \| - \| \| Abbreviations: HbA1C = glycated hemoglobin 1c; mRS = modified Rankin scale; RR = relative ratio; AR = attributable ratio.  Unless specified, values are *p* value in non-parametric tests and values are numbers of patients (%).  **^†^**Median (interquartile range).  ^*^Statistically significant. \| \| \| \| \| \| | | | | | |

**Table NOS**
